# Health consequences of disasters: Advancing disaster data science

**DOI:** 10.1093/pnasnexus/pgae211

**Published:** 2024-05-31

**Authors:** Leremy A Colf, Tony McAleavy

**Affiliations:** Fran and Earl Ziegler College of Nursing, University of Oklahoma Health Sciences Center, 1100 N. Stonewall, Oklahoma City, OK 73117, USA; Fire and Emergency Management Program, Oklahoma State University, En549, Engineering North, Stillwater, OK 74078, USA

**Keywords:** disasters, health, data

## Abstract

Understanding the health effects of disasters is critical for effective preparedness, response, recovery, and mitigation. However, research is negatively impacted by both the limited availability of disaster data and the difficulty of identifying and utilizing disaster-specific and health data sources relevant to disaster research and management. In response to numerous requests from disaster researchers, emergency managers, and operational response organizations, 73 distinct data sources at the intersection of disasters and health were compiled and categorized. These data sources generally cover the entire United States, address both disasters and health, and are available to researchers at little or no cost. Data sources are described and characterized to support improved research and guide evidence-based decision making. Current gaps and potential solutions are presented to improve disaster data collection, utilization, and dissemination.

## Introduction

A vast number of government reports, strategies, after action reports, business plans, and scientific articles, both recent and historical, have stressed the ongoing need for high-quality, accurate, broad, interoperable datasets to be used to improve decision making (1–11). These sources also highlight challenges to data collection, access, and sharing, but not limited to high costs, logistics of data collection, maintaining up-to-date data resources, privacy, and security. While progress is being made, many major challenges remain (12). The demand for data remains strong, despite estimates that the sum total of all data is increasing at 40% per year, meaning there is a doubling of data available every few years (13). With this exponential growth, particularly when much of the data consists of new types or new datasets rather than additions to existing ones, it is increasingly difficult to remain current with existing data sources or identify/engage with new sources. Subject matter experts participating in a National Academies workshop (1) identified that in many cases, the data that would be of most interest in answering key research questions already existed: rather than needing to collect those data, the more critical task was figuring out how to help researchers harness those data. They highlighted the need to determine “what is out there” and then create a central repository for data. This would serve the dual purposes of educating diverse researchers across disciplines about what data are available and also supporting them in understanding, accessing, and utilizing the already existing data sources (1).

The aim of this study is to collect and describe a wide variety of data sources at the intersection of disasters and health. The resulting compendium is designed to support both research and operational communities to conduct disaster research and implement data-driven decision making. Together, the research and operational communities can improve operational efficiency and ensure better outcomes for future emergencies, disasters, and catastrophes (collectively termed disasters herein). This study collates 73 disaster health-related data sources organized by category and theme. It also identifies areas for further data development, which were discussed at a 2024 National Academies workshop hosted by the Action Collaborative on Disaster Science (14).

## Literature review

While data demand is ubiquitous, individual disciplines are at different levels of maturity in terms of their data sharing and utilization, notably within healthcare. The sheer amount of healthcare data presents substantial challenges, exacerbated by patient privacy/protected health information concerns, the large diversity of data types, and the speed at which analysis must occur to support patient care (15). Moreover, health data are highly financially valuable, estimated at $300–$450 billion per year (13). This is a strong incentive not to share data: whoever possesses the data has a financial benefit to utilize their own data and maintain their competitive advantage, particularly when research has shown that data-driven decision making can increase productivity gains by 5%–6% (11). The editorial board of *Nature Methods* argues that the field of genomics and transcriptomics is the best model for data sharing, given the discipline's clear agreement on the types and forms of data to be shared (16). Disaster science data, by contrast, suffer from a lack of a common format and terminology, and limited data continuity, with added complications from safety and security risks for responders and data collection staff, political interests, costs, communication and cultural barriers, and limited cooperation between interested parties (1, 17). Moreover, disaster data are often ephemeral, which does not align with standard research or funding timelines. While extensive data can be collected in the response phase of a disaster, much of it is fragmented, incomplete, and inconsistent, meaning it is unsuitable for broader evidence-based decision making (7). Furthermore, data collected in a disaster lack a baseline for comparison (18), which compounds the already difficult tasks of measuring impacts, evaluating interventions, and validating recovery efforts. While these factors can be addressed through time and preparation, this can become overwhelming when addressed in a disaster response context (8). Consequently, auxiliary data must be found and used, which are often not interoperable with disaster data and/or have different standardized methodologies and definitions (6).

The need for systematic data (primary as well as auxiliary) to inform disaster preparedness and response is increasing, most notably among operational agencies. Researchers, emergency managers, and healthcare providers have repeatedly identified the need for continuous, consistent, and interoperable data before, during, and after a disaster (1, 5, 6, 8, 9). The need for data is a mix of 2 root causes: lack of data and lack of data sharing. The Federal Emergency Management Agency agrees, reporting “the current state of data sharing to be insufficient” (2). Fortunately, key stakeholders who own and control data are increasingly recognizing and prioritizing the broad societal benefits of publicly accessible data repositories. The federal government has prioritized the identification and sharing of health-related data for decades. Examples include the creation of data.gov as a public data repository in 2009 (19), and legislative provisions within the Affordable Care Act of 2010 directing the Office of the Secretary of Health and Human Services to conduct, disseminate, and expand capacity for patient-centered outcomes research and data (20). Looking forward, the federal government and disaster/health/data experts have named increasing access to federal datasets as one of their top priorities (21–23), with a specific call out for disaster data (1, 2, 4). However, there is currently no compendium of disaster or auxiliary data sources that could be used for disaster preparedness and response. Accordingly, this study addresses the research question, “What data sources exist at the intersection of disasters and health that can be used to support research?”

## Methodology

### Data source identification

Over the past decade, we have worked with disaster researchers, the health data community, the federal data community, emergency managers, and first responders to identify data sources useful for disaster research. These expert elicitations occurred from 2018 to 2023. Experts across research and operations were asked to identify data sources relevant to disasters and health at conferences and meetings. Meetings included the 2018 Preparedness Summit, the 2018 Open Geospatial Consortium Workshop, the 2019 Natural Hazards Workshop, the 2021 Patient Centered Outcomes Research Trust Fund Meeting, the National Academies Symposium on Pediatric Disaster Science, and the 2023 meeting of the Action Collaborative on Disaster Research. Participating organization/experts included the National Academies of Sciences, Engineering, and Medicine Action Collaborative on Disaster Research; the Natural Hazards Center; the American Red Cross; the Harvard Center for Climate, Health, and the Global Environment; the North American Alliance of Hazards and Disaster Research Institutes; Crisis Ready; the HHS Office of the Assistant Secretary for Preparedness and Response; and individual experts from academia, nonprofit organizations, and disaster response organizations. While the elicitation format varied, experts were provided with an introduction to the need for disaster/health data, given examples of useful data sources, presented with a research implementation example, asked what data they would most like for their research or response operations, and asked to identify additional data sources that they found useful. Hundreds of data sources were identified, ranging from broadly applicable to highly specific and specialized.

### Inclusion criteria

Inclusion decisions were informed by the authors and experts, collectively. Each data source was evaluated independently for inclusion in this compendium, according to 3 broad criteria. The criteria were also informed through the expert elicitation. When examples of useful data sources were presented to the expert groups, they identified which data sources they found useful. Many of the proposed data sources presented by one expert were identified as less valuable to other researchers. Criteria were validated with the National Academies Action Collaborative on Disaster Research and the research community. Throughout this process, 3 criteria emerged that were consistently associated with the expert-identified most valuable data sources.

Criteria for data source inclusion in the compendium:

1. Geographic focus on the United States and covering the majority of the country.
2. Relevant to both disasters and health.
3. Accessible to researchers at no or low cost.

Consequently, the majority of the data sources included in the compendium are from US federal sources. However, data sources also include private and nonprofit organizations as well as for-purchase and/or restricted data sources to illustrate the broad range of data sources available as well as providing context for the federal no-cost data. Data sources were included if they provided consistent, systematic data on a large number of disasters and would provide information on health aspects of disasters. Some examples of excluded data sources and the reason for exclusion were data collected in a single disaster that would not necessarily apply to other disasters; clinical trials data, as they generally do not distinguish between disasters and steady state; or health data that could not be tied to disasters or the time period/geography of a disaster. This selection process resulted in the inclusion of 73 distinct data sources in this compendium. A summary of these sources is included as Table 1, with additional information, links, descriptions, and a data dictionary available in a searchable format through the CONVERGE data repository and DesignSafe (24). While regional data, individual disasters, clinical trials, infectious disease outbreaks, and other data sources are highly valuable, they were not included in this compendium due to their limited scope. One of the issues preventing researchers from finding useful data sources is the sheer volume of available data. By focusing on only those data sources that are broadly relevant to the entire disaster research community, the compendium can serve as a focused, high-level resource, without having to search through a large volume of data sources that are not relevant to their research questions. Researchers interested in more focused studies are encouraged to use this compendium as a starting point and then proceed to more focused data on specific disasters/regions.

**Table 1.** Disaster science data sources.

Theme	Category 1	Category 2	Name	Source	Source type	Source subtype	Predisaster	During disaster	Postdisaster	Data type
Disaster data	Preparedness		NWS Past Weather	National Weather Service	Federal—Commerce	NOAA	Yes			Multiple
Disaster data	Preparedness		Community Resilience Estimates from Census Bureau	Census Bureau	Federal—Commerce	Census	Yes			Quantitative
Disaster data	Recovery		After-Action Report Repository	SGNL Solutions	Private				Yes	Qualitative
Disaster data	Response		Disaster mission assignment costs	FEMA	Federal—DHS	FEMA		Yes		Financial
Disaster data	Response		Public Assistance and Individuals and Households Program	FEMA	Federal—DHS	FEMA		Yes		Financial
Disaster data	Response		Disaster Declaration Summary	FEMA	Federal—DHS	FEMA		Yes		Geographic
Disaster data	Response		National Fire Incident Reporting System (NFIRS)	United States Fire Administration	Federal—DHS	FEMA		Yes		Multiple
Disaster data	Response		Disaster Research Response Resources	NIEHS	Federal—HHS	NIH		Yes		Data collection tools
Disaster data	Response		Public Health Emergency (PHE) Declarations Database	ASPR	Federal—HHS	ASPR		Yes		Geographic
Disaster data	Response		Community Assessment for Public Health Emergency Response (CASPER)	CDC	Federal—HHS	CDC		Yes		Survey
Disaster data	Response		The International Disaster database (EM-DAT)	Centre for Research on the Epidemiology of Disasters	International			Yes		Multiple
Disaster data	Response		National Shelter System	Red Cross	Nonprofit			Yes		Multiple
Disaster data	Response		Firefighter rescue survey	Firefighter Rescue Survey	Private			Yes		Survey
Disaster data	Response		COVID contact tracing	Various	Various			Yes		Geographic
Auxiliary data	Healthcare organization	Hospital	HCUP Data Tools	AHRQ	Federal—HHS	AHRQ	Yes	Yes	Yes	Multiple
Auxiliary data	Healthcare organization	Hospital	HHS Protect	HHS Protect Public Data Hub	Federal—HHS		Yes	Yes	Yes	Multiple
Auxiliary data	Healthcare organization	Hospital	Pediatric Health Information System (PHIS) database	Children's Hospital Association	Nonprofit		Yes	Yes	Yes	Multiple
Auxiliary data	Healthcare organization	Hospital	National Emergency Department Overcrowding Score (NEDOCS)	NEDOCS	Nonprofit		Yes	Yes	Yes	Quantitative
Auxiliary data	Healthcare organization	Hospital	American Hospital Association (AHA) Annual Survey	American Hospital Association	Private		Yes	Yes	Yes	Multiple
Auxiliary data	Healthcare organization	Other	HealthData.gov	HHS	Federal—HHS		Yes	Yes	Yes	Multiple
Auxiliary data	Healthcare organization	Performance metrics	Hospital Preparedness Program Performance Metrics	ASPR	Federal—HHS	ASPR	Yes	Yes	Yes	Multiple
Auxiliary data	Healthcare organization	Registry	National Trauma Databank	American College of Surgeons	Nonprofit		Yes	Yes	Yes	Registry
Auxiliary data	Healthcare organization	Surveillance	ESSENCE, the Electronic Surveillance System for the Early Notification of Community-based Epidemics	National Syndromic Surveillance Program	Federal—HHS	CDC	Yes	Yes	Yes	Multiple
Auxiliary data	Healthcare organization	Surveillance	FluView Influenza surveillance	CDC	Federal—HHS	CDC	Yes	Yes	Yes	Quantitative
Auxiliary data	Individual	Survey	Disability surveys	Census Bureau	Federal—Census	Multiple	Yes	Yes	Yes	Survey
Auxiliary data	Individual	Survey	American Community Survey (ACS)	Census Bureau	Federal—Commerce	Census	Yes	Yes	Yes	Survey
Auxiliary data	Individual	Survey	Current Population Survey (CPS)	Census Bureau	Federal—Commerce	Census	Yes	Yes	Yes	Survey
Auxiliary data	Individual	Survey	Consumer Assessment of Healthcare Providers and Systems (CAHPS)	AHRQ	Federal—HHS	AHRQ	Yes	Yes	Yes	Survey
Auxiliary data	Individual	Survey	Disability surveys	HHS	Federal—HHS	Multiple	Yes	Yes	Yes	Survey
Auxiliary data	Individual	Survey	National Survey of Drug Use and Health (NSDUH)	SAMHSA	Federal—HHS	SAMHSA	Yes	Yes	Yes	Survey
Auxiliary data	Individual	Survey	NCHS Population Surveys	NCHS	Federal—HHS	CDC	Yes	Yes	Yes	Survey
Auxiliary data	Individual	Vital statistics	National Death Index	NCHS	Federal—HHS	CDC	Yes	Yes	Yes	Multiple
Auxiliary data	Individual	Vital statistics	National Vital statistics System	NCHS	Federal—HHS	CDC	Yes	Yes	Yes	Multiple
Auxiliary data	Individual	Vital statistics	Wonder	CDC	Federal—HHS	CDC	Yes	Yes	Yes	Multiple
Auxiliary data	Individual	Vital statistics	Birth registry	CDC	Federal—HHS	CDC WONDER	Yes	Yes	Yes	Quantitative
Auxiliary data	Patient	Billing/claims	EMPOWER	ASPR/CMS	Federal—HHS	ASPR/CMS	Yes	Yes	Yes	Geographic
Auxiliary data	Patient	Billing/claims	CMS claims data	CMS	Federal—HHS	CMS	Yes	Yes	Yes	Multiple
Auxiliary data	Patient	Billing/claims	Transformed Medicaid Statistical Information System (T-MSIS)	CMS	Federal—HHS	CMS	Yes	Yes	Yes	Multiple
Auxiliary data	Patient	Billing/claims	Healthcare Cost and Utilization Project (HCUP)	AHRQ/HCUP	Federal—HHS	AHRQ	Yes	Yes	Yes	Quantitative
Auxiliary data	Patient	Billing/claims	Patient-Centered Clinical Research Network	PCORnet	Nonprofit		Yes	Yes	Yes	Multiple
Auxiliary data	Patient	Billing/claims	Blue Cross claims database	Blue Health Intelligence	Private		Yes	Yes	Yes	Multiple
Auxiliary data	Patient	Billing/claims	State specific claims databases	States	State		Yes	Yes	Yes	Multiple
Auxiliary data	Patient	Billing/claims	All payer databases	Various	State or private		Yes	Yes	Yes	Quantitative
Auxiliary data	Patient	Patient health record	Joint Health Information Exchange	DoD	Federal—DoD	DoD	Yes	Yes	Yes	Multiple
Auxiliary data	Patient	Patient health record	All of Us Research Hub	NIH	Federal—HHS	NIH	Yes	Yes	Yes	Multiple
Auxiliary data	Patient	Patient health record	National Disaster Medical System (NDMS)	ASPR	Federal—HHS	ASPR	Yes	Yes	Yes	Multiple
Auxiliary data	Patient	Patient health record	Web-based Injury Statistics Query and Reporting System (WISQARS)	NCHS	Federal—HHS	CDC	Yes	Yes	Yes	Quantitative
Auxiliary data	Patient	Patient health record	Department of Veterans Affairs Open Data Portal	Veterans Affairs	Federal—VA		Yes	Yes	Yes	Multiple
Auxiliary data	Patient	Patient health record	Health Care Systems Research Network	HCSRN	Nonprofit		Yes	Yes	Yes	Multiple
Auxiliary data	Patient	Patient health record	National Emergency Medical Services Information System (NEMSIS)	NEMSIS	Nonprofit		Yes	Yes	Yes	Multiple
Auxiliary data	Patient	Patient health record	New England Healthcare Health Information Exchange	New England Healthcare Exchange Network and Massachusetts Health Data Consortium	Nonprofit		Yes	Yes	Yes	Multiple
Auxiliary data	Patient	Patient health record	COSMOS	Epic	Private		Yes	Yes	Yes	Multiple
Auxiliary data	Patient	Patient health record	EMS Data Platform	Biospatial	Private		Yes	Yes	Yes	Multiple
Auxiliary data	Patient	Patient health record	MarketScan Commercial Claims and Encounters Database	Merative	Private		Yes	Yes	Yes	Multiple
Auxiliary data	Patient	Patient health record	Real-World Data	Cerner	Private		Yes	Yes	Yes	Multiple
Auxiliary data	Peripheral system	Community	Social Vulnerability Index (SVI)	CDC/ATSDR	Federal—HHS	CDC	Yes	Yes	Yes	Geographic
Auxiliary data	Peripheral system	Community	Sendai Framework Global Target Reporting	United Nations Office of Disaster Risk Reduction	International	United Nations	Yes	Yes	Yes	Multiple
Auxiliary data	Peripheral system	Community	USA Facts	USA Facts	Nonprofit		Yes	Yes	Yes	Multiple
Auxiliary data	Peripheral system	Community	Deloitte's Health Equity Dashboard	Deloitte	Private		Yes	Yes	Yes	Quantitative
Auxiliary data	Peripheral system	Geographic	Historical Hurricane Tracks	NOAA	Federal—Commerce	NOAA	Yes	Yes	Yes	Geographic
Auxiliary data	Peripheral system	Geographic	Superfund sites	EPA	Federal—EPA		Yes	Yes	Yes	Geographic
Auxiliary data	Peripheral system	Geographic	Healthcare Provider Shortage Areas	HRSA	Federal—HHS	HRSA	Yes	Yes	Yes	Geographic
Auxiliary data	Peripheral system	Geographic	GEOHealth	ASPR	Federal—HHS	ASPR	Yes	Yes	Yes	Multiple
Auxiliary data	Peripheral system	Geographic	CrisisReady	CrisisReady	Nonprofit		Yes	Yes	Yes	Geographic
Auxiliary data	Peripheral system	Geographic	RxOpen	Healthcare Ready	Nonprofit		Yes	Yes	Yes	Geographic
Auxiliary data	Peripheral system	Geographic	Esri Disaster Response Program	Esri	Private		Yes	Yes	Yes	Geographic
Auxiliary data	Peripheral system	Geographic	March of Dimes Maternity Care Deserts Dashboard	Deloitte	Private		Yes	Yes	Yes	Geographic
Auxiliary data	Peripheral system	Geographic	Mobility data	Cell phone and social media	Private		Yes	Yes	Yes	Geographic
Auxiliary data	Peripheral system	Geographic	Data for Good	Meta	Private		Yes	Yes	Yes	Multiple
Auxiliary data	Peripheral system	Infrastructure	Eagle-I	DoE	Federal—DoE	DoE	Yes	Yes	Yes	Geographic
Auxiliary data	Peripheral system	Infrastructure	Bloomberg intelligence	Bloomberg	Private		Yes	Yes	Yes	Multiple
Auxiliary data	Peripheral system	Other	Technical Resources, Assistance Center, and Information Exchange TRACIE	ASPR	Federal—HHS	ASPR	Yes	Yes	Yes	Multiple
Auxiliary data	Peripheral system	Survey	Occupational Employment and Wage Statistics (OEWS)	BLS	Federal—Labor	BLS	Yes	Yes	Yes	Survey

This table contains a list of 73 data sources relevant to both disasters and health that can be used by researchers and practitioners to improve research and response in disasters. The full data compendium can be accessed online (24).

Abbreviations: AHRQ = Agency for Healthcare Research and Quality; ASPR = Administration for Strategic Preparedness and Response; ATSDR = Agency for Toxic Substances and Disease Registry; BLS = Bureau of Labor Statistics; CDC = Centers for Disease Control and Prevention; CMS = Centers for Medicare and Medicaid Services; DHS = Department of Homeland Security; DoD = Department of Defense; DoE = Department of Energy; EPA = Environmental Protection Agency; FEMA = Federal Emergency Management Agency; HCSRN = Health Care Systems Research Network; HCUP = Healthcare Cost and Utilization Project; HHS = Department of Health and Human Services; HRSA = Health Resources and Services Administration; NCHS = National Center for Health Statistics; NIH = National Institutes of Health; NOAA = National Oceanic and Atmospheric Association; SAMHSA = Substance Abuse and Mental Health Services Administration; VA = Department of Veterans Affairs.

Inclusion or exclusion of data sources is not a measure of value and is not a measure of accuracy or completeness. It should also not be considered an endorsement. Rather, the compendium is intended as a starting point for researchers to identify and utilize additional broadly applicable data sources at the intersection of disasters and health. Additionally, all data are owned and managed by the organization listed as “source” in the compendium, including data security and data sharing. Researchers are encouraged to utilize the additional information and links in the online version of the compendium for more detailed information on each data source: https://doi.org/10.17603/ds2-vjre-t833

### Data source categories and themes

The 73 data sources are organized by categories and themes and a brief description of each source is included to guide researchers to new data. Identification, categorization, and description were challenging given the breadth and diversity of the available data sources. While decisions were made in good faith, adherence to inclusion and exclusion criteria means that the compendium is fundamentally subjective and incomplete. The categorization, descriptions, and links were accurate at the time of writing, but may change. Further, additional, and emerging data sources will likely warrant future inclusion.

Data sources were organized by theme, as either disaster data or as auxiliary data, in which auxiliary is defined as containing information about the disaster geography or time period but are secondary data for the disaster. Per the inclusion criteria, each data source must sit at the intersection of disasters and health. Grouping the data sources by category and theme enables identification of more and lesser represented data areas. The disaster science community can therefore use these categorizations as a starting point to collectively discuss opportunities to improve disaster data, and identify areas for collaboration with data, policy, and response communities to better utilize auxiliary data.

### Findings

**Fig. 1.**
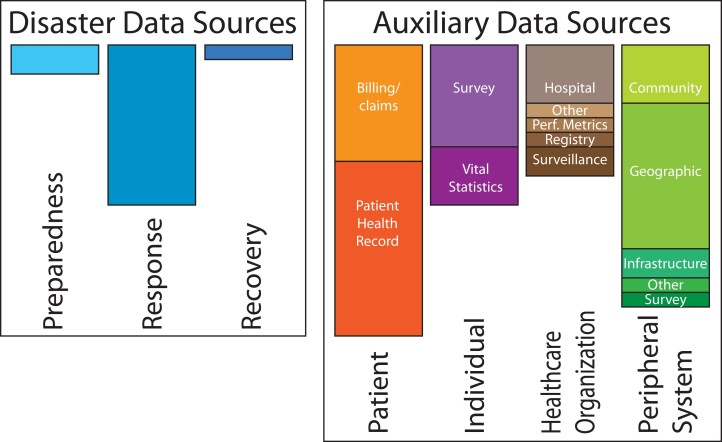
Data source themes and categories. This figure visually represents the distribution of the 73 disaster health data sources identified within this study. The size of each bar is proportionally scaled to the number of data sources within each theme (disaster data sources or auxiliary data sources) and category, with the actual numbers and percentages described in the Findings and available in the data compendium.

As described in Table 1 and summarized in Figure 1:

- *Disaster data*: 19% of the data sources (14 of 73) were classified as disaster data, including 2 preparedness, 11 response, and 1 recovery data source. The majority of preparedness and response data sources are federal, more so than the auxiliary data sources.
- *Auxiliary data*: the remaining 81% of data sources (59 of 73) were classified as auxiliary data, including 10 healthcare organization, 11 individual, 20 patient, and 18 peripheral system sources. The greater proportion of auxiliary data sources is not surprising, given that all data sources not primarily disaster data but related to disasters and health are considered auxiliary.

Disaster data sources can be further categorized according to the time period(s) covered by the data. In general, disasters are temporally described according to the phases of the disaster management cycle (i.e. mitigation, preparedness, response, and recovery), but these phases can overlap temporally (e.g. Federal Emergency Management Agency hazard mitigation grants are awarded during the disaster recovery period, but as preparation for future disasters), which would result in ambiguous classification. Data sources were, therefore, categorized as comprising data covering the predisaster period, period amid the disaster, and/or the postdisaster period (Table 1, columns 8–10). Under this classification, all auxiliary data sources spanned the entire cycle, with continuous data across the predisaster, during disaster, and postdisaster periods. Each disaster data source, by contrast, covered only a single time period. This lack of continuity in disaster data presents a significant challenge for the disaster science discipline. If data are limited to the duration of disaster, it is near impossible to determine an accurate pre-event baseline to measure consequences. It is similarly challenging to determine when recovery is complete (i.e. a return to baseline), or to evaluate the effectiveness of preparedness or other interventions without data from pre-event, during event, and postevent, as each of these research processes requires at least 2 time periods of data, if not all 3.

Because the auxiliary data sources included in this compendium do not fit into distinct disaster time periods, they are instead categorized according to the origin of the data. This will better allow researchers to understand the type of data included in each source. In some cases, the source of the data is not the same as the origin of the data. For example, claims data originate from a healthcare facility where a patient is treated. However, when those claims data are provided by an insurance company, the source is no longer a healthcare provider. Furthermore, when those data are part of mandatory reporting to the federal government, or are collected by a third party, the source can be even more disparate. However, in all cases, billing and claims data originate with the patient. Similarly, nonhealth data originate with an individual, whether they are part of a survey, vital statistics repositories, or other sources. Some data do not originate with a patient or individual, but instead arise from an organization or system, such as hospital performance metrics or community-level data. These are categorized as originating from a healthcare organization (hospitals, nonprofits, or government organizations primarily focused on health) or peripheral systems (organizations providing data that may be useful for disaster and health research but are not primarily healthcare focused). This categorization helps researchers understand the origin and type of data contained in each data source, regardless of the type of organization providing data access.

Categorizing data temporally for disaster data and by origin for auxiliary data supports the identification of challenges and opportunities in the disaster science data discipline. Figure 1 represents the relative prevalence of each data type in this compendium, with an increased size for more prevalent categories. While this compendium is not exhaustive, there are some potential insights from source prevalence:

- Disaster data are less prevalent than auxiliary data. This is to be expected, given that auxiliary data can draw from any field that potentially overlaps with disasters. However, it does highlight that there are very few disaster data sources and highlights the challenges of data collection in a disaster. The disaster science community may want to consider ways to increase collection, access, and utilization of additional primary data.
- Preparedness and recovery data are less prevalent than response data. Within disaster data, response data constitute the vast majority, with nearly 3 times as many sources as preparedness and recovery data combined. The National Academies has previously highlighted the importance of collecting and sharing standardized baseline data (1).
- Healthcare organization data are less prevalent than peripheral system data. This is to be expected, given that healthcare organization data is a limited field definition, while peripheral system data could draw from any field or discipline. Instead, this highlights the value of using other data sources and of learning from other fields in order to strengthen and improve disaster data science.
- Patient data are more prevalent than individual data, although this is likely an artifact of inclusion criteria. Patient data are, by definition, health data and are therefore more likely to apply to disasters and health than more general individual or community-level data such as surveys or registries.
- There are very few surveillance data sources included in this compendium. In the COVID-19 era, disease surveillance is receiving increased focus, particularly for the challenges of identifying new and emerging infectious diseases or disorders. However, this is likely the result of selection criteria rather than the existence—or lack—of data sources.

In addition to categorizing data sources by theme (Table 1, column 1), category (Table 1, columns 2–3) and the organization providing access to the data (Table 1, columns 5–7), data sources were also organized by the type of data they contain (Table 1, column 11). Most of the data did not fit clearly into standard statistical variable categorization (e.g. nominal, ordinal, continuous, etc.). More specifically, the data sources are made up of large relational databases in which the data simultaneously consisted of too many data types for simple typing. Instead, data sources were typed to help researchers identify broad categories of data such as geographic, financial, survey, qualitative, or quantitative. Even with these broad categories, 36 of the 73 data sources consisted of multiple data types. This is not surprising, given that the inclusion criteria required overlap of different types of data sources. Interestingly, qualitative data such as surveys were highly represented—11 data sources in addition to qualitative components of the 36 described previously—despite expectations that most nationwide data sources would be numerical (e.g. quantitative, financial). Notably, 14 data sources were typed as geographic mapping data, in contrast to reports in the literature that geographic disaster databases were lacking (8), indicating advancement of the discipline over the past decade. The high prevalence of qualitative data allows for social science and mixed methods research that can help inform why things occur, not just describe effects.

Analysis of data source availability and prevalence led to identification of 4 main challenges:

1. Data collection in a disaster
2. The value of using other data sources in disaster research
3. Learning from other fields
4. The challenge of surveillance

## Discussion

### Data collection in a disaster

Ideally, data would be continuously collected on everything, everywhere, at all times. Unfortunately, this presents substantial feasibility and financial challenges. Predicting when and where a disaster would occur and collecting baseline data in that location could address these challenges. However, the capacity to accurately predict disasters far in advance is limited, meaning that such an approach, while useful, would be problematic. Alternatively, rapid data collection systems could be established to capture data during and following a disaster, particularly temporal, transient, or ephemeral data. While these approaches can be successful for individual incidents, they are not necessarily representative: different data elements are collected, the data are not interoperable, and often only the analysis is made available, rather than the underlying data. Thus, while these efforts are individually valuable, the community should focus on high-level continuous data while individual research teams focus on incident-specific data.

### The value of using other data sources

Any individual data source or data type will likely not address all aspects of a disaster. It is therefore important to consider a wide range of data sources in research and response/operations. For example, disaster data sources address only a single time period (before, during, or after the disaster), whereas billing/claims data provide continuous data. Researchers can use these continuous data to determine the baseline for the number of individuals seeking care at a specific hospital and then measure the change in hospital utilization during the response and recovery phases of a disaster. They can also determine when the hospital and the region have recovered by measuring the return to the predisaster baseline. Additionally, researchers could compare interventions used at other, similar hospitals to see if those interventions had an effect on utilization, patient outcomes, or the recovery time frame. Without the context provided by predisaster, during disaster, and postdisaster data, it is challenging (if not impossible) to clearly define the nature of the problems facing the disaster health community, identify potential improvements, or measure progress. Alternatively, auxiliary data sources are also unlikely to address all aspects of a disaster. The auxiliary data sources included in this compendium will likely require extensive cleaning and modification in order to make disparate datasets interoperable. Additionally, these auxiliary data come from different fields, often have different definitions of key terms, lack valuable fields or variables, or do not address key portions of the affected population. The community should, therefore, focus on building relationships with leaders in auxiliary fields and developing a shared lexicon and data format to make data sources interoperable and more useful for disasters. As an additional benefit, these approaches could also improve the utility of data collected in a disaster.

### Learning from other fields

Disaster data sources make up a 19% minority of sources included in this compendium, whereas health data and data describing the community (e.g. geographic, demographic, socioeconomic) are more prevalent and often more mature in their organization, complexity, and maturity. This presents an opportunity for the disaster science data community to learn from other fields regarding how to build and optimize data resources, how to find and incorporate auxiliary data, how to work with relevant communities to share data resources, and how to develop tools to improve interoperability. Given the lack of available data sources, it can be challenging to make data-driven decisions that best support communities impacted by disasters, including response, recovery, and emergency management organizations. It is only through improving data resources and utilizing those resources that the disaster science community can identify and implement ways to better support impacted individuals, communities, and systems. As the disaster science community works to develop and utilize additional data resources, they can look to other more mature data disciplines for best practices in data management, collection, and organization.

### The challenge of surveillance

Disease surveillance databases are minimally represented in this compendium. This low representation is likely the result of selection criteria, rather than the lack of data sources. Infectious disease data sources are a potentially interesting area to explore, particularly given that disease surveillance can provide early warnings for and ongoing information on outbreaks, pandemics, or bioterrorism attacks. However, given that these infectious disease events require surveillance and rapid response—often involving novel, emerging, or re-emerging infectious agents—they are less suited to large existing datasets and more suited to surveillance and anomaly detection. Disease surveillance through data systems has many of the same challenges as disaster science data, including data quality, privacy and security, and interoperability (25), and may be relevant for a limited period of time and only during the outbreak/epidemic, rather than as a continuous data source like the other auxiliary data described in this compendium. By comparison, there is a plethora of chronic disease data sources, including the CMS Chronic Conditions Data Warehouse and disease-specific registries and data sources (e.g. diabetes, heart disease, and some cancers). Most chronic condition data sources, however, are focused on health but lack a specific disaster component. While they could be very useful for some disaster research questions, they are not so broadly applicable as to warrant inclusion in this compendium. However, it could be useful in the future to examine chronic conditions that are the result of disasters, such as the World Trade Center Health Registry or the recent discussions of registries to determine the long-term consequences of wildfire smoke inhalation (26).

It is interesting to speculate on the overrepresentation of data owned/provided by the federal government that is categorized as disaster data sources compared with auxiliary data. A total of 64%–75% of disaster data are from federal sources (depending on if federal means United States exclusively or federal more broadly), whereas only 57% of auxiliary data are from federal sources. One possible reason is the challenges with private or nonprofit disaster data collection. Unless they are part of the operational response, private or nonprofit organizations may not be involved or welcome to collect data in the response. It is also challenging for individual organizations to collect nationally representative data, as it requires ubiquitous presence and national reach. Another possible reason is the lack of financial resources for ongoing data cleaning, hosting, and sharing among nonfederal stakeholders. Finally, there may not be a business case for collecting or sharing disaster data. The clear potential economic gains from other types of data have incentivized private sector growth of open data (27). Health data, for example, have a high commercial value for disease and target identification, drug development, process improvement, and other business use cases. One recent estimate valued data-based healthcare improvements as potentially saving $300–$450 billion per year in reduced healthcare spending (13). Perhaps there are fewer business value propositions for disaster data, and there is therefore a need to incentivize the private sector to share data for the benefit of the whole of the community (28). Or perhaps those value propositions are not prioritized due to competing interests, failing infrastructure, scarce resources, shareholder concerns, the federal deficit, and other concerns that impact disaster, data, and health policy.

### Themes and issues in data source usage

Disaster science is rather young in comparison with other more established disciplines such as mathematics, medicine, and the traditional sciences (29). Given this relative modernity, the scope of the discipline, which is grounded in anthropology, geography, sociology, and psychology, is loosely defined and open to researcher interpretation (30). Furthermore, the data sources, terminology, and processes of new disciplines are generally underdeveloped, lacking, or a mixture of incompatible legacies from other disciplines. The choices that both academic and practitioner researchers make regarding the importation, utility, and validity of data sources often reflects their disciplinary backgrounds (31). Throughout the process of compiling and categorizing disaster science data sources, we identified 2 critical data management issues, namely the inclusion of both qualitative and quantitative data, and interoperability, which must be addressed in order to improve future data collection, sharing, and utilization.

### Quantitative or qualitative data sources

Research and policy are sometimes erroneously believed to be more numerically based, which inadvertently places greater emphasis on quantitative as opposed to qualitative data sources due in part to the perceived objective certainty of numbers (32). Disaster science is, however, grounded in exploratory applied qualitative studies, which informed the early development of the discipline (33). Both quantitative and qualitative methods have merit depending on the nature of the researcher's specific focus and the questions to be addressed (34). The distinction between the 2 types of data is often reduced to a matter of numbers versus words. However, differentiation is more nuanced, as some quantitative data can be more artificial when informed by existing theory and concerned with measuring behavior, whereas qualitative data are more descriptive, naturalistic, emergent, and focused on meaning. The differentiation is, therefore, a question of measuring behaviors or describing meanings, rather than simply numbers or words. A researchers choice of data source should, therefore, be determined by the needs of their research question, rather than individual, institutional, or funder predisposition (31).

Disaster science has evolved from exploratory qualitative postdisaster field studies to incorporate quantitative, multimethod, and mixed methods studies, meaning that disaster science–informed policy can, and should, incorporate both quantitative and qualitative data sources (33, 35). This can be challenging, however, when one of the inclusion criteria for data sources in this compendium was coverage across the entire United States. Quantitative data often have a common definition and scale and can thus be compared across disasters and geographies more easily. Qualitative data provide feeling, context, and impact, but generalizability is often limited as a result of distinct communities (e.g. geographies, cultures, or disaster types). Thus, while highly valuable and insightful, not all qualitative datasets met the criteria for inclusion in this compendium. Included data sources were of 2 types: nationwide surveys, especially those with ordinal categories or Likert scales; or mixed type datasets that link qualitative (e.g. descriptive, demographic, or socioeconomic) data with quantitative data. For example, billing and claims data combine the number of disaster-related patient health claims with demographic data on the impacted individuals. These mixed datasets allow careful examination of the underlying causes for disaster vulnerability and resilience. Regardless, there is a clear role for both qualitative and quantitative research in disaster science. Every disaster is local, and the impacted individuals and their lived experiences cannot be ignored, nor can the measurable impacts of numerical data. Given the historical bias toward quantitative data and the emotional bias toward qualitative data, it is noteworthy that several data sources in this compendium incorporated both qualitative and quantitative data, linked and interoperable in ways that can provide context to the numbers.

### Interoperability: Technology, people, and processes

The interoperability of disparate data sources is a critical issue in disaster data science, as well as in data science in general. Interoperability (particularly in a nascent discipline such as disaster science) is about much more than simply linking datasets: it is more than a technological problem as it integrates concern about the people and processes that utilize the technology (36). The term *interoperability* is, however, understood differently across disciplines. In 2021, the US Office of the National Coordinator for Health Information Technology engaged both the public and health systems leadership to describe what interoperability meant to them (37). Definitions from the public ranged from accessible data to access to care to the roles of healthcare providers. Health systems definitions ranged from compatible technologies to linked data to continuous and clear data across the disaster life cycle. The 21st Century Cures Act defines interoperability in statute: “The term ‘interoperability’, with respect to health information technology, means such health information technology that enables the secure exchange of electronic health information with, and use of electronic health information from, other health information technology without special effort on the part of the user; allows for complete access, exchange, and use of all electronically accessible health information for authorized use under applicable State or Federal law; and does not constitute information blocking” (38). The Department of Homeland Security describes the interoperability challenge as ensuring “anyone can talk with whomever they need to, whenever they need to, when properly authorized” (39, 40). The scientific literature defines interoperability in terms of stakeholders being able to work together (41, 42), and as a markedly broader concept with various definitions that transect technological, people and, procedural contexts (36). With such wide-ranging and potentially disagreeing definitions of interoperability, it is unsurprising that distinct fields struggle to achieve interoperability.

In terms of disaster science data, understanding of interoperability is wide-ranging and is perhaps better defined by the questions interoperable data can help answer. These include: “What common data elements need to be collected in a disaster to produce useful, reliable health data that can be compared across populations and disasters?”; “How can datasets and data sources be merged to continuously cover pre-, during, and postdisaster periods?”; “How can the health consequences of disasters be measured and compared?”; “How does the disaster science community make data accessible and available to those who need it in a disaster?”; “How do researchers and data managers ensure patient privacy is maintained in data sharing?”. Interoperability sits at the core of (almost) all issues of disasters and health. It is a multidimensional concept that incorporates technology, training, decision making, policies, procedures, language, culture, subculture, norms and values, and more (41). Accordingly, the noted themes and issues relating to data source utility are explored herein using Cole's interoperability framework, which emphasizes technology, people, and processes to ensure that researchers can effectively utilize the broad range of available data sources.

### Technology

Interoperability is most commonly framed in a technological guise; indeed, the Emergency Management Institute (43) emphasizes the ability of systems, personnel, and equipment to exchange data and information. Historic efforts to build interoperability have understandably focused on the compatibility of equipment and technology (36). The Healthcare Information and Management Systems Society (44) states that interoperability works at 3 distinct levels: foundational, structural, and semantic.

1. Foundational interoperability is the base level of technological interoperability whereby the constituent systems can readily exchange data and information but cannot interpret it, meaning that understanding is reliant on researcher knowledge, skill, and experience.
2. Structural interoperability is considered a higher level and refers to a state whereby systems can freely exchange data and information, which is interpreted at the data field level.
3. Semantic interoperability is considered the highest achievable level and refers to a state whereby multiple systems can freely exchange data and information, which affords greater utility.

The terms *integration* and *compatibility* are often erroneously used as synonyms for interoperability which hampers conceptual lucidity. Technological interoperability requires both integration and compatibility, but they are not one in the same, as they are distinct but interconnected concepts. Integration refers to the process of combining multiple systems so that they talk and operate as one while maintaining compatibility on an ongoing basis. Compatibility, however, relates to the ability of systems to interact concurrently in the same environment and perform their intended tasks without compromising the operation of the other system. This does not require interoperability as there is no need for the systems to talk and interact with one another (45). Jardim and Martins (46) emphasized the ability of systems to communicate and exchange data in their conceptualization of technical interoperability. The communication and exchange of data does not, however, ensure understanding as interoperability beyond systems integration and compatibility requires more than technology (47). The development and adoption of the HL7 Fast Healthcare Interoperability Resources standard has been instrumental in defining how healthcare data can be exchanged across technology systems, regardless of how it is stored in those systems. It does not, however, address data definitions or common data elements upstream of storing and sharing. This is a gap between the nascent needs in disaster science health data and the established norms of the healthcare community.

### People

Interoperability is vital within disaster response as much as in disaster data: the more interoperable stakeholders are, the more likely they are to be able to work together in an effective manner (41, 42). Per the Department of Homeland Security’s interoperability definition cited previously, they must include over 60,000 federal, state, tribal, and local stakeholders in pursuance of the whole of the community approach (39, 40).

Severson (48) stated that interoperability refers to “the ability of emergency response personnel to interact & work together.” While logical, this perspective overemphasizes the role of emergency response personnel, negates the broader requirement for whole of the community engagement, and actively excludes the many other disciplines involved in disaster science (49). Indeed, it could potentially violate interoperability by constituting information blocking as defined in the 21st Century Cures Act (38). Alternatively, the United Kingdom's Joint Emergency Services Interoperability Principles, known as JESIP, states that interoperability is “the extent to which organizations can work together coherently as a matter of routine” (50). This broader perspective affords greater scope to integrate technology as well as people and processes in the pursuit of data source interoperability (36, 47). In other words, interoperable data sources and technology are useless if the whole of community does not understand, value, and utilize the data. Furthermore, the data must be applied with the correct understanding and context to avoid the philosophical argument that numbers can lie discussed previously (51).

Disaster science is a broad discipline that transects the natural, social, and applied sciences in a combined effort to increase the capacity of the whole community to prepare for, respond to, and recover from emergencies, disasters, and catastrophes (52, 53). The resultant social melting pot consists of several disciplinary backgrounds with distinct cultures, subcultures, norms, and values that present a significant barrier to the advancement of disaster science that cannot be overcome with technological interoperability alone (54). A researcher's culture, subculture(s), norms, and values are guided by ideas, meanings, beliefs, feelings, and actions that inform decisions about whom to work with and what data sources are valid and should be used (55). Culture can be taught, learned, or simply picked up through exposure: it is an important mechanism that enables a researcher to fit in by developing an applied understanding of appropriate behavioral patterns (56). Individual disciplines within disaster science have distinct formal cultures and several covert or clandestine subcultures, which may be integrated, differentiated or fragmented which hampers interoperability (57, 58). Researchers must, therefore, maintain a level of cultural awareness, as their disciplinary background, education, training, and institution will inform their research design, either overtly or covertly, notably their philosophical and methodological disposition and data type and source preferences (31). Interoperability requires technology to facilitate access to and exchange of data. Understanding, however, requires applied technological competence to access data and scientific literacy to identify research gaps, utilize the data, and draw empirical conclusions. Researcher cultural intelligence can augment technological competence and scientific literacy by enabling the researcher to interact more effectively with a broader range of diverse stakeholders, which can promote multi- and interdisciplinary research to advance disaster science (56, 59). Technological competence, scientific literacy, and cultural intelligence are, therefore, critical; however, the framework of processes in which these elements operate can either help or hinder interoperability creating unintended barriers.

### Processes

Interoperability between technological systems or between groups of people is relatively straightforward and easy to understand. The role of process in interoperability, however, may be less clear. Policies, regulations, procedures, and standards at the disciplinary and inter- and intrainstitutional level can either enable or hinder a researcher's ability to access, understand, and utilize data effectively (36). Processes also direct data collection, storage, and sharing meaning they can help bridge the interoperability gap by enabling data to exist and be shared in a timely and effective manner (39, 40). Policies establish clear procedures for what types of data can be shared, the circumstances under which they can be shared, and how and in what format (60). Moreover, the standardization of technical specifications, content, and vocabulary within and across data sources can promote effective utilization by enhancing data source utility (61). However, process is more than just the framework for interoperability. It also forms the first principles that allow emergent disciplines (i.e. disaster science) to build interoperability into the definition of their organizations and into the habits of their researchers and practitioners. Processes and policies become a shared social contract between people that is built into the technology. They define the core data elements that will be collected in a disaster and establish the cross-disciplinary agreements that researchers and practitioners will collect the same data in the same ways. They also establish data use and data sharing agreements that will promote research while simultaneously protecting patient privacy. Greater focus on the technical foundation and the cultural norms and values that underpin interoperability are necessary to enhance data source design and utilization (62). In disaster science, the overlap of multiple disparate parent disciplines leads to inherent conflict of processes and policies. First responder organizations emphasize the need to reduce the use of agency-specific codes, jargon, and both technical and colloquial language in order to enhance communications and promote interoperability via the development of shared meanings and understanding (63). Healthcare data, by contrast, notably patient medical records and billing data, rely on agency-specific codes and technical language for accuracy and specificity. Indeed, reimbursement regulations require Centers for Medicare and Medicaid Services–specific codes and technical language and researchers rely on these codes (64), putting their needs and norms at direct odds with the needs of first-responder organizations. How, then, can the discipline establish interoperability between different people and technology systems? Only through the hard work of collectively developing policies and processes that are in the best interest of all.

## Conclusions

This compendium presents 73 data sources relevant to the intersection between disasters and health to address one of the major issues repeatedly identified within disaster science. Namely, informing researchers from a wide variety of backgrounds as to what data sources exist to support academic and operational research (1–11). Neither the compendium of data sources nor the broader study, however, solves the many other data issues affecting disaster researchers. Given the exponential increases in data each year (13), the compendium will quickly become obsolete. However, the rapid increase in data also presents an opportunity. Given the relative newness of disaster science, the number of data sources is still small. By collecting what is available now, the disaster science community can identify strengths, weaknesses, and opportunities and address them before weaknesses become entrenched. Together, the community can also look to other disciplines and employ technology leapfrogging to build a better approach to data collection and data sharing.

While the stated aim was to compile and describe data sources at the intersection of disasters and health, this study also identifies several opportunities that may be of interest to the discipline. These future opportunities arise from analysis of the available data sources, including their categories and themes, as well as comparison of these data sources with those available in other disciplines.

Five conclusions that inform both research and practice are the following.

1. There are very few disaster health data sources.

A total of 73 data sources were identified at the intersection of disasters and health. In contrast, at the time of writing, the Institute for Health Metrics and Evaluation identified 9,185 health data sources for the United States in their Global Health Data Exchange with additional data sources added regularly (65). By comparison, there is a paucity of disaster science sources, meaning that there is substantial room for improvement in developing and sharing additional data sources.

2. Disaster data are less represented than auxiliary data.

Data sources were grouped into themes and categories. Analysis of the data source distribution across the categories suggests an underrepresentation of disaster data, notably data sources pertaining to the preparedness and recovery phases of the disaster management cycle. This stems from the well-established health and other auxiliary data fields and is reflective of the inherently uncertain nature of disasters, which necessitates a focus on life, infrastructure, and the environment (i.e. saving lives), rather than data collection. There is a growing desire for disaster data due, in part, to increasing societal hypercomplexity and more frequent, devastating, and costly disasters that necessitate more data to inform understanding throughout all phases of the cycle (66, 67). This suggests that the data, disaster, and health communities should prioritize collection, compilation, and dissemination of disaster data. Collecting and sharing this type of data should be a government priority. Mechanisms should also be explored to incentivize private and nonprofit organizations to collect and share disaster data.

3. Disaster data sources are generally limited to one time period.

Collecting predisaster baseline data or establishing data collection strategies before, during, and after a disaster can be problematic. However, disaster science research requires continuous data for evaluation of efficacy, measuring recovery, and improving patient outcomes in disaster healthcare delivery. Researchers should, therefore, explore ways to utilize continuous auxiliary data sources to establish these baselines and better inform disaster research. Policy and evaluation experts should explore surrogate measures for preparedness.

4. Interoperability is lacking.

Interoperability is understood in different ways though it is generally lacking according to all definitions. The disaster science community has a unique opportunity to bring together relevant stakeholders to define common data elements, establish data sharing and data use agreements, and support the collection of interoperable data.

5. Leapfrogging is a viable path forward.

The concept of technology leapfrogging, whereby a nation bypasses traditional development steps and jumps directly to the state of the art developed by another, can be applied to disaster science as a less developed field by looking to more established fields for inspiration. This could include the adoption of standards and norms from healthcare or emergency management in ways that benefit all stakeholders. While leapfrogging is not as common in disaster response as in other disciplines, there is precedent for technology leapfrogging in disaster data collection and data sharing (68).

## Data Availability

The data compendium described in this manuscript is available online and is maintained by Converge and DesignSafe-CI. Colf, L., T. McAleavy. (2023) “Compendium of data sources addressing disasters and health.” DesignSafe-CI. https://doi.org/10.17603/ds2-vjre-t833
